# A Case of Typhlitis Stercoralis

**Published:** 1888-05

**Authors:** S. L. Post

**Affiliations:** Gainsville, Cook Co,, Texas


### A CASE OF TYPHLITIS STERCORALIS.

*By S. L. Post, M. D., Gainsville, Cook Co,, Texas.*

For Daniel's Texas Medical Journal.

TYPHLITIS, strictly speaking as we have it from our best scientists, is a disease limited to affections of the cæcum and its appendix vermiformis, and its allied affections or complications show especial predilection for the male sex, although the researches of some of our best scientists show that the disease pre-eminently affects the bloom of life. One of our contributors to medical literature, of eminence in the scientific investigation of this disease, claims that affections of the vermiform appendix have hitherto received only step-motherly treatment at the hands of the anatomists and clinicians (Krassald), as anatomy shows that the walls of this organ are so sparsely endowed with muscular tissue as to render it unable to expel any foreign accumulation that might enter it through the comparatively stagnant reservoir, the cæcum. This fact being known, with the comparative uselessness of this structure has led some eminent medical men to consider the vermiform appendix with the significant appellation of a "death trap."

However, as I feel unable to attempt to throw any light on this affection which is only of modern recognition, I will proceed to report the case that came under my observation, as it might interest some reader of your JOURNAL.

Mr. S., lawyer by profession, came to my office April 6th. to consult me in reference to a severe pain that he had been suffering with for some time, located in right illiac region. Upon examination by palpation and percussion, I very soon came to the conclusion that I had a typical case of typhelitis, or an impaction in the vermiform appendix. Mr.S. informed me that while in Austin, April 1st, attending to some legal business, and during the time that he was making an argument on a case he had before the court, he was attacked with a very acute pain in the right illiac region, the pain increasing so rapidly that he was unable to finish his speech. Consequently sought medical aid for the alleviation of the agonizing pain that he was suffering. After opiates were administered, and other treatment given him by his medical attendant, necessary for the time being, he returned home, with an occasional paroxysmal pain in the right illiac region. I at once suggested irrigation of the bowel, first using warm water and castile soap, removing at each evacuation small quantities of hard fecal matter and mucus. After several irrigations I decided it might be a better plan to use an oleaginous preparation as a lubricator. I slipped over the end of an Alpha syringe tube an English rubber catheter. Oiling it well, I passed it up to the arch of the colon, throwing through the gum catheter a ten grain solution muriate coeaine, waiting fully twenty minutes before injecting the olive oil. The bowel now being thoroughly cocainized, I placed the patient in the knee and chest position, in order that the oil might come in direct contact with the inpaction. Having everything in readiness, I began gently injecting the oil, using fully three quarts of warm olive oil, at a temperature of about 90 degrees F. I had no trouble in getting in the bowel a sufficient quantity to distend the organ beyond its then present capacity, and to myself and patient's greatest satisfaction the evacuation of the oil, after remaining in the bowel fully thirty-five or forty minutes, was followed by large quantities of grape seeds and preserved citron and English peas, seemingly in a natural state of preservation. Patient cannot call to memory eating grapes since last spring, 1887, nor any English peas since last fall, but ate heartily of fruit cake last Christmas, containing citron, with the other necessary ingredients usually in such luxuries. Patient now is convalescing rapidly, and all irritation in the locality of the appendix vermiformis is giving away. The above treatment, with an occasional dose of mag. sulphas, grs. xx. was all I did.

